# A Bimodal Model to Estimate Dynamic Metropolitan Population by Mobile Phone Data

**DOI:** 10.3390/s18103431

**Published:** 2018-10-12

**Authors:** Jie Feng, Yong Li, Fengli Xu, Depeng Jin

**Affiliations:** Department of Electronic Engineering, Tsinghua University, Beijing 100084, China; feng-j16@mails.tsinghua.edu.cn (J.F.); xfl15@mails.tsinghua.edu.cn (F.X.); jindp@tsinghua.edu.cn (D.J.)

**Keywords:** bimodal model, real-time metropolitan population estimation, mobile phone data

## Abstract

Accurate, real-time and fine-spatial population distribution is crucial for urban planning, government management, and advertisement promotion. Limited by technics and tools, we rely on the census to obtain this information in the past, which is coarse and costly. The popularity of mobile phones gives us a new opportunity to investigate population estimation. However, real-time and accurate population estimation is still a challenging problem because of the coarse localization and complicated user behaviors. With the help of the passively collected human mobility and locations from the mobile networks including call detail records and mobility management signals, we develop a bimodal model beyond the prior work to better estimate real-time population distribution at metropolitan scales. We discuss how the estimation interval, space granularity, and data type will influence the estimation accuracy, and find the data collected from the mobility management signals with the 30 min estimation interval performs better which reduces the population estimation error by 30% in terms of Root Mean Square Error (RMSE). These results show us the great potential of using bimodal model and mobile phone data to estimate real-time population distribution.

## 1. Introduction

Knowing real-time population distribution around the city is important for many fields, such as urban planning, business location, transportation schedule and especially emergency management. If the government knew the real-time population distribution in Shanghai Bund on 31 December 2014 and took action on the crowd, many innocent tourists may have escaped the tragedy. Despite its great value, there is no mature system, even a reliable model, to accomplish this task perfectly up to now. In the past, the census, with great cost in money and clerks, is still the main method to obtain population distribution. Traditional methods usually use the satellite images with dasymetric modeling tools to estimate population distribution [1]. Furthermore, as the state of the art works [2], researchers can obtain the population distribution by using random forest model to estimate population from satellite images. Even though, they can only update the results once a month which is far from the real-time application.

In the past decade, with the popularity of mobile networks and mobile phones, we have the opportunity to observe human mobility around the city from an entirely new viewpoint. Many researchers have done some great works on human mobility at the individual level. Gonzalez [3] and Song [4,5] use mobile phone data to prove that human mobility is regular in the most of the time. Isaacman [6,7], Ficek [8] and Jiang [9] build models to describe and predict human mobility. Wu [10] and Krings [11] pay attention to human communication and find prevalent communication laws. Calabrese [12,13] uses mobile phone data to sense city and the transportation system. At high levels, researchers even try to understand people’s life style [14] and friendship structures [15].

Researchers also explore human mobility in the aggregate level, e.g., crowd detection and population estimation. For the irregular group mobility, Dobra [16] and Dong [17] build effective detection systems to infer the unusual crowd events based on the mobile phone data. In this work, we focus on the real-time population estimation. Given the assumption that call records density can approximate to human density to some extent, Isaacman [7] builds a human mobility model-WHERE at metropolitan scales. Obtaining the population results by aggregating the individual estimation trajectory, the accuracy and efficiency of the WHERE model are poor. In 2014, Deville [18] proposes a model based on the widely used power law to estimate population. This work accustoms a great step forward of the traditional work at both space and time granularity. However, because of the limit of temporal and spatial granularity, it cannot generate real-time population distribution during the day or precise population distribution within the minimum administrate unit. To estimate the real-time population based on cellular data accessing logs, Xu [19] develops an extended model based on the simple power law by taking regions’ function into account. Douglass [20] also uses the power law model to obtain the real population based on the mobile phone data in Italy, he observes that distribution of the population is bimodal. In addition, Wu [10] says that the bimodal distribution in short message is the natural result of interacting human activities. Up to now, previous works consider only part of the underlying factors, no one have taken the human interact and various mobile phone data together into account in the previous work.

In this paper, inspired by the evidence of bimodal power law from previous work, we extend the simple power law model with more suitable data to the bimodal model to estimate the real-time population at the street blocks level. Here, we use road networks to generate the street blocks. We set the methods proposed by Xu [19] as our baseline. Moreover, we discuss the performance of different type, temporal and spatial granularity of cellular data on our model. We find that the cellular data collected from Mobility Management with 30 min interval outperforms others. The cross evaluation results reveal that our model can obtain the real-time population distribution with the estimation error reduced by 30% compared with the state of the art.

## 2. Material and Methods

### 2.1. The Bimodal Model

**Figure 1:**
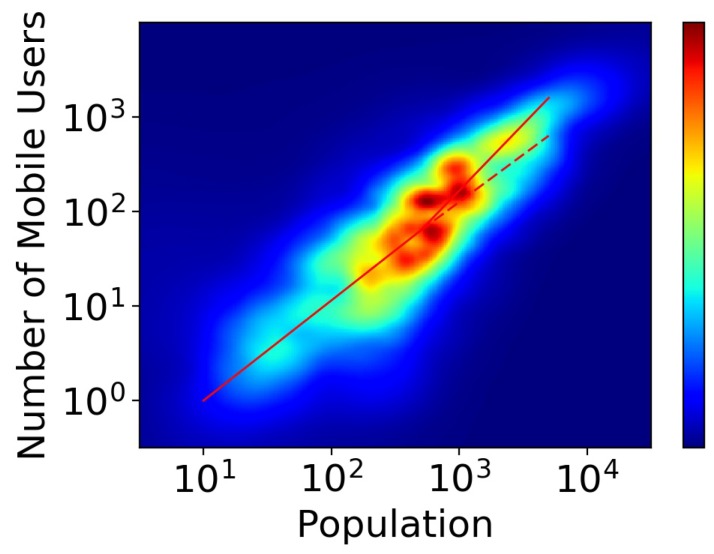
The linear correlation between log-scale mobile phone users and log-scale population.

As Figure 1 shows, the linear correlation between log-scale population and log-scale number of mobile phone users seems obvious. However, when we pay attention to the part with more users and population, it seems that they follow different rules compared to the part with less users and population. Thus we propose a bimodal power law model to better estimate population distribution, which is expressed as follow,

$${\hat{p}}_{i}=\left\{\begin{array}{cc} \alpha_{1}{\left(u_{i}\right)}^{\beta_{1}} & u_{i}<\theta ,\\ \alpha_{2}{\left(u_{i}\right)}^{\beta_{2}} & u_{i}>=\theta , \end{array}\right.$$

where ${\hat{p}}_{i}$ is the estimated population of region *i*, $u_{i}$ is the mobile users or the mobile phone logs, α represents the scale factor and β represents the super-linear effect involved by the mobile users’ behaviors. Specifically, we set θ as a threshold to divide regions into two classes by the number of mobile phone users. In our model, the region is not the smallest administrative unit [18] or a prevalent grid [20]. For better semantic representation, we need meaningful region representation methods. Yuan [21] use road network to describe regions. Space syntax [22], which is widely used in traditional Geographic Information System (GIS) field, is another choice. Considering the scalability and explanatory, we choose regions enclosed by the road network as the space units.

So why is the amount of users the point? We may explain that by considering the human behaviors in different regions. As the idea popular in city planning and sociology says, the greater the population the greater the communication and activity level. With more chances to meet face-to-face, people have fewer chances to communicate with each other by mobile phone. As a result, we need adjust parameters to meet this gap. From the view of telecom infrastructures, we can also obtain the similar result. If people in the region with high population density use their phone just like people in the region with lower population density, they are faced with more risks on network problem which can restrain their mobile using behaviors. In a word, people will change their mobile phone usage when there are too many people around them.

Since Xu [19] finds people in different functional regions have various phone usages like frequency of accessing network, regions’ function should also be considered as a crucial parameter in this problem. Besides, we adjust α to meet the real-time demands given that α acts as a scale factor. After considering these, we obtain a context-aware model as follows,

$${{\hat{p}}^{t}}_{f^{j}}=\left\{\begin{array}{cc} \alpha_{j}^{t}{\left(u_{f^{j}}^{t}\right)}^{\beta_{j}} & u_{f^{j}}^{t}<\theta ,\\ \alpha_{j}^{t}{\left(u_{f^{j}}^{t}\right)}^{\beta_{j}} & u_{f^{j}}^{t}>=\theta , \end{array}\right.$$

where suffix *f* is a zone collection with the same function, superscript *j* is function label, superscript *t* is the time index. ${{\hat{p}}^{t}}_{f^{j}}$ and ${u^{t}}_{f^{j}}$ represent the estimated population and the number of mobile phone users in a certain area belonging to zone collection $f^{j}$ whose function label is *j*. In the piecewise equation, α and β represent the parameters for regions whose users less than the threshold θ, α and β belongs to regions with more users and people. The threshold θ is decided by parameter searching in the training stage. If the amount of users in an area is less than θ, we will use the first equation to fit and estimate the population of it. Otherwise, the second equation is used for estimation.

### 2.2. Data Description

**Figure 2:**
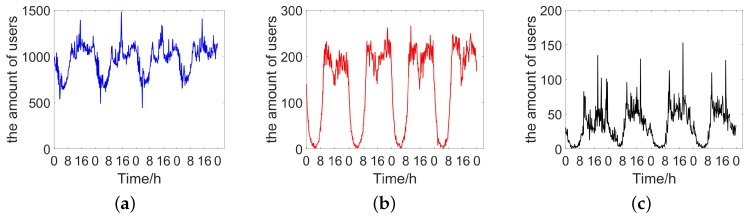
The amount of users extracted from three types datasets in a sample region. (**a**) The amount of users extracted from the mobility management signals in a sampl region; (**b**) The amount of users extracted from the call detail records in a sample region; (**c**) The amount of users extracted from the short message records in a sample region.

**Figure 3:**
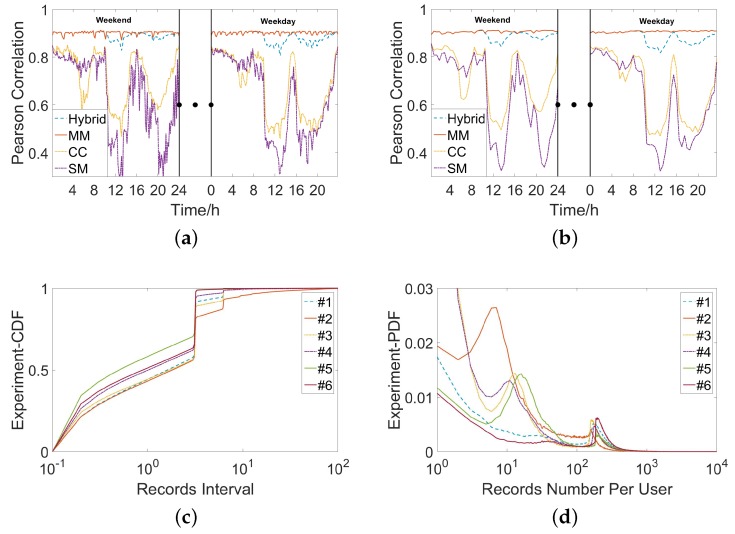
The characteristics of three kinds of data in six cities. The record number of each user is calculated by summing up the number of records belonging to him in the whole data. Here, MM denotes mobility management signals data, SM denotes short message records, CC denotes call detail records, and Hybrid denotes the mixture of three kinds of data. (**a**) With setting the counting time slot as 10 min, the correlation between the mobile phone users and the population among all the regions in Urlumqi; (**b**) With setting the counting time slot as 30 min, the correlation between the users and the population among all the regions in Urlumqi; (**c**) The records interval distribution in 6 cities; (**d**) The records number of single user distribution in 6 cities.

The dataset is collected by the operator in the cell tower level at the second week of July on 2016. The dataset contains three kinds of data: mobility management signals, short message records and call detail records. Mobility management data is the data generated when cellular network tries to call up user devices with a fixed period to provide seamless inter-working. In the experiment, we also call the mixture of three kinds of data as hybrid data. The dataset covers 6 cities in Xinjiang China. All the records are anonymous by replacing the phone number with a unique ID. There are many business attributes in the collected datasets, but only spatial-temporal characteristics are needed in our problem. Thus, with necessary data cleaning, each cleaned record only consists of four attributes: time stamp, cell tower id, user id, and its service type. Figure 2 presents the amount of users extracted from three types datasets in a sample region. The details of characteristics of data are presented in Figure 3. According to Figure 3, 90% of the records interval are less than 30 min and 60% users have at least 200 records. Identical with Deville [18], we choose WorldPop project [2] as our static ground-truth. WorldPop project provides estimates of numbers of people residing in grid cell around the world, which is used as the basic data in many projects. In addition, the data is updated once a month.

### 2.3. City Segmentation

We choose street blocks enclosed by road network as our basic space analysis unit. This is because that human activities generate the street blocks and most blocks are self-contained function unit. Besides, the street blocks are flexible in size. We obtain the road network image with the help of Baidu Map Application Program Interface (API) [23]. Then we use morphological methods to obtain the street blocks.

**Figure 4:**
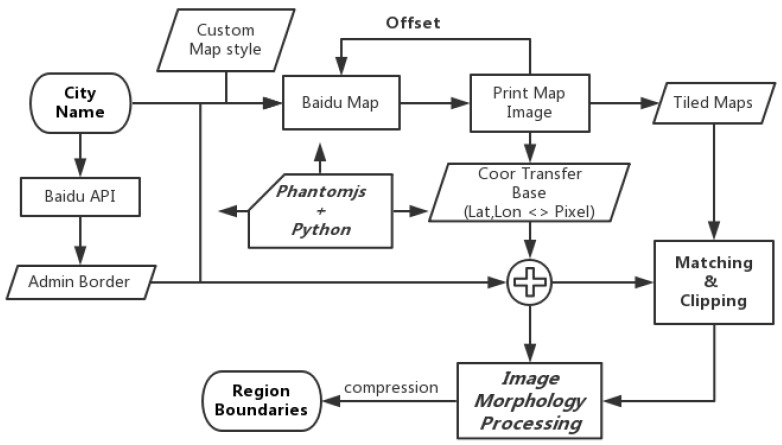
Framework for download map data from the Baidu Map API.

**Figure 5:**
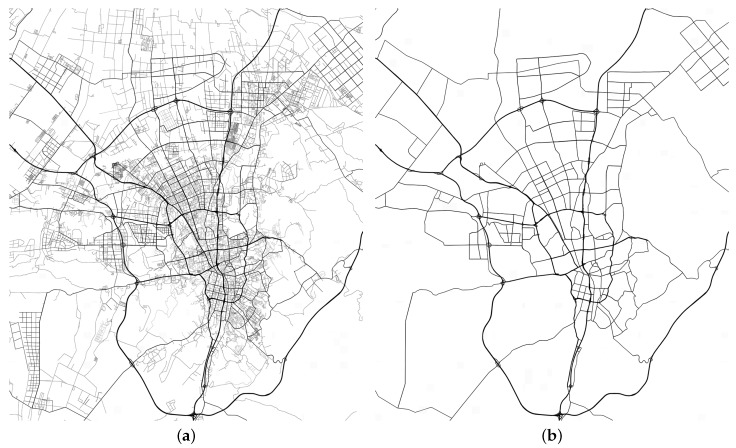
The road network map of Urlumqi. (**a**) L1 road network map of Urlumqi; (**b**) L2 road network map of Urlumqi.

Spatial granularity is crucial for obtaining the meaningful and accuracy population estimation results. Most previous works did not pay enough attention to it and they chose the grids or admin units because they are simple enough. When we scan the whole city, we find that the road network is import for human mobility and regions enclosed by it is a natural segmentation of the city. Thanks to the road network, the size of the region is approximately proportional to its distance to the center of the city and its population, which is a very useful character for us. So we choose the regions enclosed by the road network as our spatial granularity. In addition, now the question is how to complete this. Because of the lack of road network data, we design a system to get it from the Internet with the open access. Baidu Map [23], who opens parts of its substantial map data to developers and researchers, allows us to custom the whole map to leave the road network alone. Based on this custom road network map accessing on the web, we design a typical framework to download the raw data and process it. Our framework is showed in Figure 4. In our system, the input is the city name and the map level, the output is the road network map and some necessary information for further image morphology processing. We first custom the base map to only show the road network which is necessary for the further processing. By accessing another Baidu Map API, we obtain the boundaries of the city to lead the next download step. Then, the core step of our system is to download the whole road network with a certain order and some necessary coordinate information. We may directly download the whole map one time, but some problem stop us to do so because the whole map with enough precision is too big to be printed by the web browser. Although after setting the map to the proper size so that Safari can print the big map, we will face with the data missing problem caused by the big data volume and the poor network accessing and finally obtain the useless map. To work out this problem, we divide the whole map to several parts and identify them by id like the tile map in the web GIS system. In this way, we can download these slices parallel and record their vertex coordinates in the terms of the earth coordinates and projection coordinates. Thanks to this download method, we obtain a valuable table which can be used to transfer the projection coordinates of the road to its earth coordinates. We build this auto-download system with the help of PhantomJS [24] and Python [25]. We have opened this code in our lab web site [26], anyone that has interest can obtain it very easily. Now, we obtain the whole road network map and know how to get the corresponding Global Position System (GPS) coordinates for every pixel of it. We show an example of road network map in Urumqi in Figure 5. Particularly, we define 3 kinds of road network: L1 (expressway), L2 (major avenue), L3 (side road).

**Figure 6:**
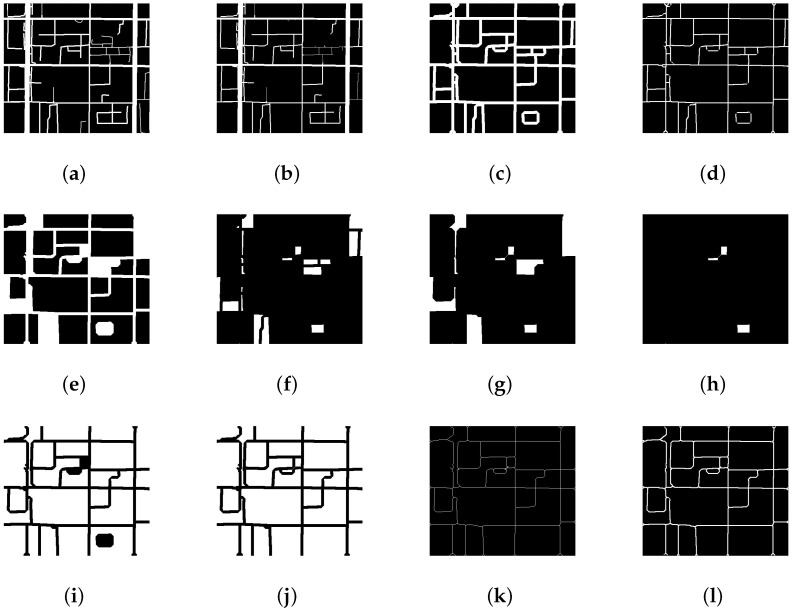
Details of city segmentation by road map. (**a**) Binary image of road network of a local region; (**b**) Apply erode operator and open operator to remove alleyways; (**c**) Apply dilate operator to smooth the residual of alleyways; (**d**) Apply thin operator to obtain the skeleton of the road network; (**e**) Calculate region area and remove the small regions; (**f**) The small regions removed by last step; (**g**) Apply dilate operator to the small regions to link the near regions; (**h**) The small regions removed finally; (**i**) Add the expansive smaller regions back; (**j**) Apply thin operator to obtain the skeleton of the road network again; (**k**) Apply dilate operator to smooth the road residual; (**l**) Add the expansive small regions back.

Based on the road network map obtained by our auto-download system, we use an raster-based model to represent the road network and apply some image morphologies to cut the whole city into many street blocks we need. In raster model, binary image is the basic object, e.g., “0” means road segments and “1” represent the regions. From the raw binary road network image, we first apply dilation operator to the road segments to fill the small hole and smooth the lane of road. Then the thin operator is applied to the road segments to obtain the skeleton of the whole road network. Considering the regional function, we can label the near regions with the same function with the same id and merge them to a big one. As for identifying the function of the specific regions, we use the simple K-means method to cluster the regions’ POI (point of interest) distribution which is normalized by term frequency–inverse document frequency (TF-IDF). If we do not care about or cannot obtain the function label, the former step can be ignored. In addition to merging the near small regions by function label, we can also consider using the user or the population amount as the label to merge the small regions together. When merging several small regions to a big one, we should check if these small regions stretch over the advanced road. The merging step should be stopped if the crossing happens, we should reduce the small regions to ensure the merging step will not produce a strange crossing region which may hurt the functional independence of the regions. In the final step, by applying edge detection algorithms like canny edge detection, we obtain the boundaries of the region. By now, we finally obtain the region boundaries from only one city name input. More details about these steps are shown in Figure 6. In these figures, we use some widely used jargons in image morphology area like thin, close, and open operator. These jargons are only alias of combination of two basic operator, dilate and erode, you can learn them easily from any image textbooks. Table 1 records the amount of regions in 6 cities at different spatial level. After the map data is ready, we come to discuss the data fusion.

### 2.4. Data Fusion

**Figure 7:**
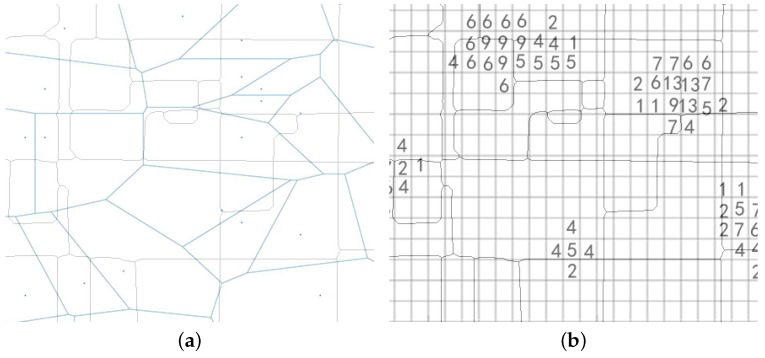
Core steps for mapping users from voronoi polygons to grid cells. (**a**) Map the users from base station to the street region; (**b**) Map the population from WorldPop to the street region.

As mentioned before, we use the WorldPop project as ground truth to calculate and evaluate the population estimation. However, neither the mobile phone records or the WorldPop population can be directly mapped into our regions. In WorldPop project, the population distribution is published in the pixel format whose spatial granularity is 100 m × 100 m grid as declared. As for the mobile phone data, its location is identified by the cell tower ID whose coverage area can be describe by the Voronoi polygons. We need to transfer both of them to the street block level. Figure 7 shows the main idea to complete this transformation. We calculate the overlapping area between the base station and the street block, then we redistribute the users of base station to the street block according to the overlapping ratio. As for the WorldPop project, one street block usually contains several 100 m grids, so we regard the accumulation population from these grids as the population of the street block. When a grid belongs to two or more regions, we partition its population to these regions according to the overlapping ratio. After aggregating the user records in the street block level, we know how the number of users vary with time in the regions like Figure 2 shows. Now, data fusion is ready and we have completed all the data preparing work.

### 2.5. RMSE and Correlation

We choose normalized root mean square error (RMSE) and correlation coefficient as performance metrics. We can calculate them as the following,

(1) $$\left\{\begin{array}{c} \varepsilon =\frac{\sqrt{\frac{1}{F}\sum_{i=1}^{F}{\left({\hat{p}}_{i}^{t}-p_{i}\right)}^{2}}}{\frac{1}{F}\sum_{i=1}^{F}p_{i}},\\ C=\frac{\sum_{i=1}^{F}\left({\hat{p}}_{i}-\frac{1}{F}\sum_{i=1}^{F}{\hat{p}}_{i}\right)\left(p_{i}-\frac{1}{F}\sum_{i=1}^{F}p_{i}\right)}{\sqrt{\sum_{i=1}^{F}{\left({\hat{p}}_{i}-\frac{1}{F}\sum_{i=1}^{F}{\hat{p}}_{i}\right)}^{2}}\sqrt{\sum_{i=1}^{F}{\left(p_{i}-\frac{1}{F}\sum_{i=1}^{F}p_{i}\right)}^{2}}}, \end{array}\right.$$

where ε represents RMSE, *C* means person correlation coefficient. *F* is the zone collection, $p_{i}$ is the ground-truth population of region *i* and ${\hat{p}}_{i}$ is the estimation results of region *i*.

## 3. Results

### 3.1. Cross Evaluation of Population Estimation

We use widely used metrics RMSE and correlation to evaluate our population estimation results. First, we show the performance gain because of the usage of the bimodal model. Then, we focus on three factors, which may play an important role in the evaluation, include data type, time span, and space granularity. To simplify the performance analysis and highlight the point, we ignore the difference between different regional functions in the following analysis and if not declared the city we investigate is just Urumqi. To stress the core factor, there are some default settings on data processing: time slot equals 10 min, space granularity is the smallest street block and default data is the mobility management data. We choose box-plot as the main presentation tool. For every moment, we calculate RMSE and correlation for the city and we draw all of them in one box. In this way, we can ensure our analysis indeed captures the full view of results rather than just something like a snapshot. Here baseline model means the simple power-law model with only one fitting curve.

**Figure 8:**
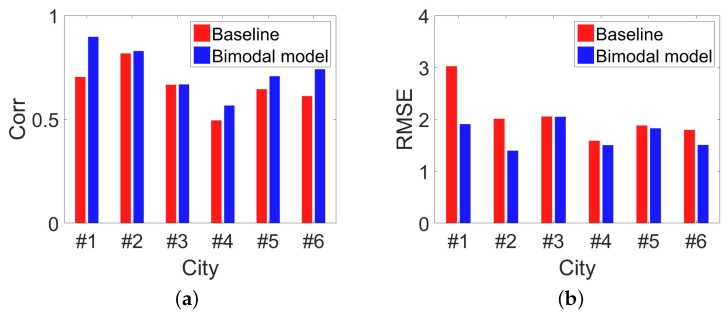
Precision assessments of baseline model and bimodal model. We can see that bimodal model performs better than the baseline model in all the cities. (**a**) Correlation between estimated population with ground-truth among 6 cities by baseline model and bimodal model; (**b**) RMSE of estimated population for baseline model and bimodal model in 6 cities.

**Figure 9:**
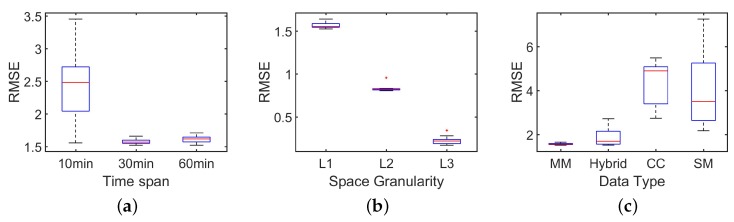
Accuracy evaluation based on RMSE. (**a**) RMSE with different time slot; (**b**) RMSE at different space granularity: L1 (the smallest space unit), L2, L3 (administrate units); (**c**) RMSE with different data: MM (mobility management data), CC (call records), SM (short message logs).

**Figure 10:**
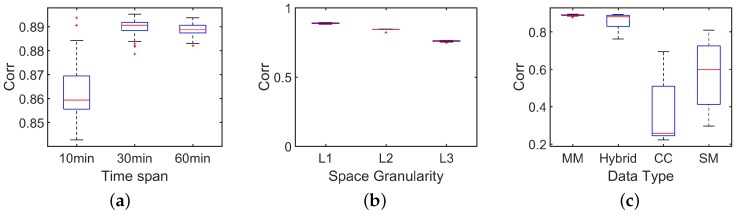
Accuracy evaluation based on correlation. (**a**) correlation with different time slot; (**b**) correlation at different space granularity: L1(the smallest space unit), L2, L3 (administrate units); (**c**) correlation with different data: MM (mobility management data), CC (call records), SM (short message logs).

As Figure 8 shows, compared with the simple power-law model, bimodal model improves the estimation performance on RMSE by about 30%. Based on the observation that mobile users behave differently among regions with different population densities, our bimodal model indeed captures the “lost” people behind the missing mobile phone records. In Figure 9a and Figure 10a, we investigate how the time span between two estimations will influence the estimation performance. Based on our dataset, we choose three time slot, i.e., 10 min, 30 min, 60 min. For example, by setting time slot of 10 min, we estimate population distribution every 10 min. As Figure 9a and Figure 10a show, RMSE goes down and correlation comes up when the time slot increases, which really satisfy our expectation. When time slot increase from 10 min to 30 min, the correlation is increased by 6% and the normalized RMSE is decreased by 40%. This result is obvious because when time slot is increasing, we have more chance to capture these inactive mobile users. There is a tradeoff between estimated accuracy with time scales. Proper time accuracy should be chosen for the specific issues and required accuracy. Next, we observe the influence involved by the different space granularity.

**Figure 11:**
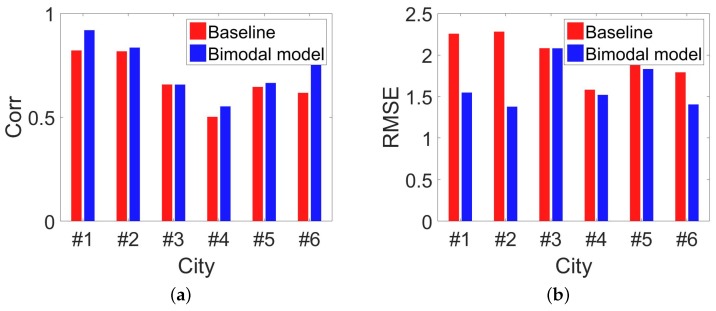
Only use mobility management signals and set time slot as 30 min which is 10 min in the main paper. (**a**) Correlation between estimated population with ground-truth among 6 cities by baseline model and bimodal model; (**b**) RMSE of estimated population for baseline model and bimodal model in 6 cities.

As mentioned before, our regions are street blocks enclosed by road networks. When we combine arterial roads with local roads to segment the city, we obtain space granularity named L1, which is the smallest street block. When just using arterial roads, we obtain space granularity named L2. Finally, the administrative unit is the third space granularity named L3. For Figure 9b and Figure 10b, we set time slot as 10 min. When we set another time slot, the main results keeps and the full view of the results can also be obtained. From Figure 9b, we can observe evident down of RMSE with the space granularity is becoming coarse. When space granularity is changed from L1 to L2, the normalized RMSE is decreased by 13%. With bigger regions, more people stay in it and randomness of human behaviors are hidden and it is natural to see the decreasing RMSE. Surprised, we find the correlation is declining slightly. In our paper, we choose RMSE as our main metrics and use correlation. Figure 11 shows the accuracy comparison between our bimodal model and baseline model when the time slot is 30 min. This slightly declining tells us that correlation is not always a good measurement in this problem.

### 3.2. The Effects of Data Type

The next question is what kind of data achieves the best performance in the estimation. There are three kinds of mobile phone data, i.e., mobility management signals, short message records and call detail records. When combining these three kinds of data together, we call them hybrid data. Call detail records and short message records are generated when mobile users use these services. Mobility management signals is a little different which is suit for population estimation. There are two scenarios for mobile users to generate mobility management signals. The first mode is that the mobile phone replies to the periodic enquiry from the base station. Secondly, when mobile users move out of the coverage of the present cell tower or logical area and move into the coverage of the new one, mobility management mechanism can also be activated. Figure 9c and Figure 10c show us the performance of every kind of data on the condition with 10 min time slot and L1 space granularity. We can observe from Figure 9c and Figure 10c that the mobility management signals shows the best performance with the lowest RMSE and the highest correlation. Generally, short message records works better than call detail records. Through, call detail records are more stable in the most of the time. It is not surprised about this result when we recall how these data are generated. In mobility management signals, 80% of the intervals between records are no more than 31 min with about 40% of the intervals are exactly 30 min. Besides, the volume of the mobility management signals is about 10 times of the volume of call detail records and short message records. Because of its high frequency and stable recording, the mobility management signals stands out from three kinds of data.

### 3.3. Interesting Observations

**Figure 12:**
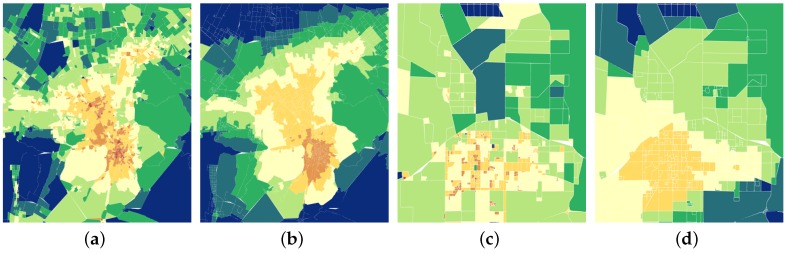
Comparison of estimated population density with baseline density for Urumqi and Shihezi. We use MATLAB [27] R2015b to generate the map-style image. (**a**) Estimated Population distribution in Urumqi. (**b**) Baseline population distribution in Urumqi. (**c**) Estimated Population distribution in Shihezi. (**d**) Baseline population distribution in Shihezi.

Based on our real-time population estimation results, we are able to analyze the insight about city dynamics. Now, we introduce human density visualization and estimate the intra-city and inter-city activity. To visualize the human density, we first map the population of every region to 8 levels by a logarithmic function. Then, every level is corresponding to one color and the red color means more people while the green one means fewer people. We show Figure 12 with comparing our population estimation results in Urumqi and Shihezi with the WorldPop project. Obviously, estimation results tell us more details about the population distribution. Our model not only works well on the big city like Urumqi but also on the small city like Shihezi, which gives us much confidence on the effectiveness of our model and data.

**Figure 13:**
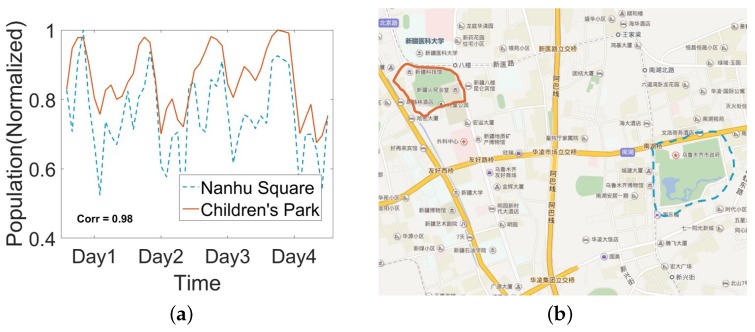
(**a**) Population flow trend for two particular regions. The time slot is 2 h. (**b**) The locations of two particular regions. MATLAB 2015b is used to draw the borderline on the Baidu Map web page.

**Figure 14:**
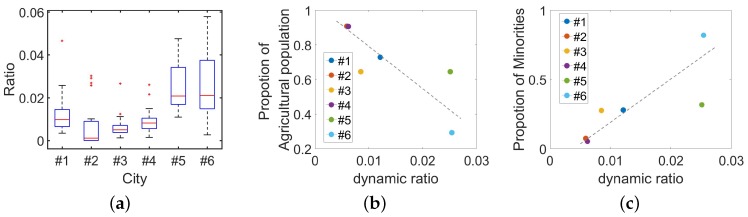
(**a**) Population flow dynamic index in 6 cities. (**b**) Relation between population composition (the proportion of agricultural population) and population flow dynamic index. (**c**) Relation between population composition (the proportion of minorities) and population flow dynamic index.

From the estimation results, we are able to analyze the intra-city activity by the population variability of regions. For example, we find there are two regions with similar population flow patterns as Figure 13a shows. By drawing two regions on the map, we observe that two regions are not near to each other. However, their functions are similar, both of them contains a park and some government agencies. In this way, we obtain the ability to understand the population flow in the city. Now, let us pay attention to inter-city dynamics. Generally, population flow can reveal the dynamics [13,28]. Thus, we calculate the proportion of regions whose population variation exceeds threshold during the morning as the dynamic index. The fluctuation can be captured by the standard deviation of the flowing population. For every region in the city, we calculate the std of its flowing population. Then, we count how many regions reach the local std threshold, which is defined as the average population variation around the city. We draw the results from six cities in Figure 14. From the results, we can find an underlying trend that bigger city with more people can be more dynamic from Figure 14a. However, we also find there are two outliers. Therefore, maybe the total population is not the only point. To find the underlying factors, we consider some other social-economic factors like the GDP, the composition of the economics and the composition of the population. All of these factors can be obtained from census data. After regression analysis, we confirm the composition of the population plays an important role in the city dynamics, including the ratio of agricultural population and the ratio of the ethnic minority. In addition, we show the regression analysis results in Figure 14b,c. From the results shown in Figure 14b,c, we can observe that more ethnic minorities and less agricultural population can account for the trend to some extent. We think maybe different lifestyles that exist in the population groups are the key points.

## 4. Discussion

In the analysis above, the bimodal model based on the mobile phone data shows great potential on the problem of population estimation. To the best of our knowledge, mobile phone data is the best data to understand the large-scale human daily lives. Instead of its coarse spatial and temporal granularity, mobile phone data is collected by the operator with low cost and cover almost everyone. Because of these great advantages, increasing researchers from different fields throw themselves to this area.

Based on the bimodal model, we can know where people are at any time, understand the human mobility and sense the pulse of the city. Our bimodal model is tested on 6 cities by four kinds of data. The evaluation results show that the performance of the model is corresponding to the city size and the source data type. Our model works better in bigger cities. Data with fine temporal granularity also improves the model performance. Moreover, we need to expand our model for small cities and other data sources, which can be enhanced by considering the interaction between regions.

According to the limited experiment results from regions with different function, we obtain similar observations to Xu et. al. [19]: the population of residential and entertainment areas with stronger periodic regularities are more correlated with their function, while the population from industry areas are more irregular and with lower correlation. Because of the lack of POI data, we could not do further analysis with enough confidential evidence on how the region’s function will influence the population estimation results in detail. We did not compare the performance of mobility management data and data accessing records in the same city by the same model. Because of its complexity, our real-time scheme is just based on the real-time input. These problems are left as the future work.

## 5. Conclusions

In this paper, we develop a bimodal model beyond the prior work to better estimate real-time population distribution at metropolitan scales. We discuss how the estimation interval, space granularity, and data type will influence the estimation accuracy. According to the experiments, we find that the periodical mobility management data is the most suitable data for population estimation. Furthermore, with more and more coarse-grained time and space granularity, the estimation error of population can be reduced a lot. However, by taking the requirement from realistic applications into consideration, we suggest that the proper time granularity is half an hour and the practical space granularity is the region enclosed by the arterial level (L2) road network. The results from experiments show us the great potential of using bimodal model and mobile phone data to estimate real-time population distribution. In future, we plan to apply the model in small cities and other data sources (such as POI information) to achieve more general and explainable results.

## Figures and Tables

**Table 1:** The number of regions in six cities at three different spatial levels. To obtain the L1 level city segmentation, we use the freeways, city expressways, and the urban arterial roads to partition the city. As for L2 level city segmentation, we remove the urban arterial roads. L3 level city segmentations equal to the administrate units.

Items	#1	#2	#3	#4	#5	#6
L1	2358	298	540	449	337	507
L2	311	26	22	37	6	36
L3	12	-	-	-	-	-
